# Evaluating and Enhancing Large Language Models’ Performance in Domain-Specific Medicine: Development and Usability Study With DocOA

**DOI:** 10.2196/58158

**Published:** 2024-07-22

**Authors:** Xi Chen, Li Wang, MingKe You, WeiZhi Liu, Yu Fu, Jie Xu, Shaoting Zhang, Gang Chen, Kang Li, Jian Li

**Affiliations:** 1 Sports Medicine Center, West China Hospital, Sichuan University Chengdu China; 2 Department of Orthopedics and Orthopedic Research Institute, West China Hospital, Sichuan University Chengdu China; 3 West China Hospital, West China School of Medicine, Sichuan University Chengdu China; 4 Shanghai Artificial Intelligence Laboratory OpenMedLab Shanghai China; 5 West China Biomedical Big Data Center, West China Hospital, Sichuan University Chengdu China; 6 Med-X Center for Informatics, Sichuan University Chengdu China

**Keywords:** large language model, retrieval-augmented generation, domain-specific benchmark framework, osteoarthritis management

## Abstract

**Background:**

The efficacy of large language models (LLMs) in domain-specific medicine, particularly for managing complex diseases such as osteoarthritis (OA), remains largely unexplored.

**Objective:**

This study focused on evaluating and enhancing the clinical capabilities and explainability of LLMs in specific domains, using OA management as a case study.

**Methods:**

A domain-specific benchmark framework was developed to evaluate LLMs across a spectrum from domain-specific knowledge to clinical applications in real-world clinical scenarios. DocOA, a specialized LLM designed for OA management integrating retrieval-augmented generation and instructional prompts, was developed. It can identify the clinical evidence upon which its answers are based through retrieval-augmented generation, thereby demonstrating the explainability of those answers. The study compared the performance of GPT-3.5, GPT-4, and a specialized assistant, DocOA, using objective and human evaluations.

**Results:**

Results showed that general LLMs such as GPT-3.5 and GPT-4 were less effective in the specialized domain of OA management, particularly in providing personalized treatment recommendations. However, DocOA showed significant improvements.

**Conclusions:**

This study introduces a novel benchmark framework that assesses the domain-specific abilities of LLMs in multiple aspects, highlights the limitations of generalized LLMs in clinical contexts, and demonstrates the potential of tailored approaches for developing domain-specific medical LLMs.

## Introduction

The rapid development of large language models (LLMs) has shown promising potential in the medical field, as demonstrated by their ability to pass the United States Medical Licensing Examination and diagnose clinical conditions [1-3]. The promising performance of LLMs in the general medical field warrants further research and exploration of their clinical performance in domain-specific medical scenarios [4,5].

Osteoarthritis (OA) is one of the most prevalent and debilitating diseases that causes pain, disability, and loss of function [6]. The global prevalence of OA is approximately 7.6% (595 million people) as of 2020 [7]. The management of OA requires complex strategies that encompass a variety of pharmacological treatments, lifestyle alterations, rehabilitation, and surgical interventions across multiple disciplines. Effective management of this condition necessitates the integration of extensive evidence-based medical data and the consideration of individual circumstances [6].

Although some LLMs have achieved commendable results in general medical question-answer (QA) tasks, substantial limitations persist in their clinical capability, particularly in complex and multifaceted diseases such as OA [8]. However, the data sets used to train LLMs are predominantly composed of general medical knowledge and lack in-depth, domain-specific content. Existing research indicates that current training data and benchmarking methodologies may be inadequate for LLMs to acquire the necessary domain-specific knowledge and clinical capabilities [4].

Additionally, LLMs may lack the ability to translate their knowledge into clinical proficiency. Despite possessing sound knowledge about certain diseases, effectively applying this knowledge to disease diagnosis remains challenging for LLMs [9]. This observation highlights the need to train and evaluate LLMs using data sets that are more closely aligned with clinical applications, thereby bridging the gap between theoretical knowledge and practical clinical usage.

To address these challenges, we proposed to build a data set that focuses on specific medical diseases, which should encompass updated evidence-based medical knowledge capable of providing both physicians and patients with expert disease-related information. In addition, real-world cases featuring patient information and treatment decisions encountered in clinical practice should be included. This repository can serve as a benchmark for testing the performance of LLMs in specific medical domains, such as OA management.

Moreover, despite the fact that LLMs have demonstrated impressive capabilities, their internal mechanisms remain unclear. This lack of transparency poses unnecessary risks to downstream applications [10], which is particularly crucial in the medical field and constitutes a significant ethical consideration [11]. Retrieval-augmented generation (RAG) offers a solution for explainability, as the RAG technique enables large models to identify the source of their answers when responding to questions. RAG is an AI framework that improves LLMs by integrating relevant information from external knowledge bases, thus enhancing the accuracy and reliability of the model's responses while also providing efficient and cost-effective access to updated external data [12]. Therefore, the integration of RAG and prompt engineering could enable the model to assimilate external knowledge bases and adhere to instructions to respond in a predetermined manner.

In general, we propose a data set framework that encompasses updated evidence-based medical knowledge, and real-world cases may effectively examine the capabilities of LLMs in clinical practice. The integration of RAG and prompt engineering may allow trained LLMs such as GPT-4 to acquire domain-specific abilities. Moreover, the management of OA serves as an ideal example in terms of its clinical significance and data volume on this research topic. Therefore, this study aimed to curate a data set for OA management, evaluate knowledge of updated evidence-based medicine for LLMs and their capabilities in clinical scenarios, and adopt RAG and instruction prompts to enhance these capabilities.

## Methods

### Overview

**Figure 1:**
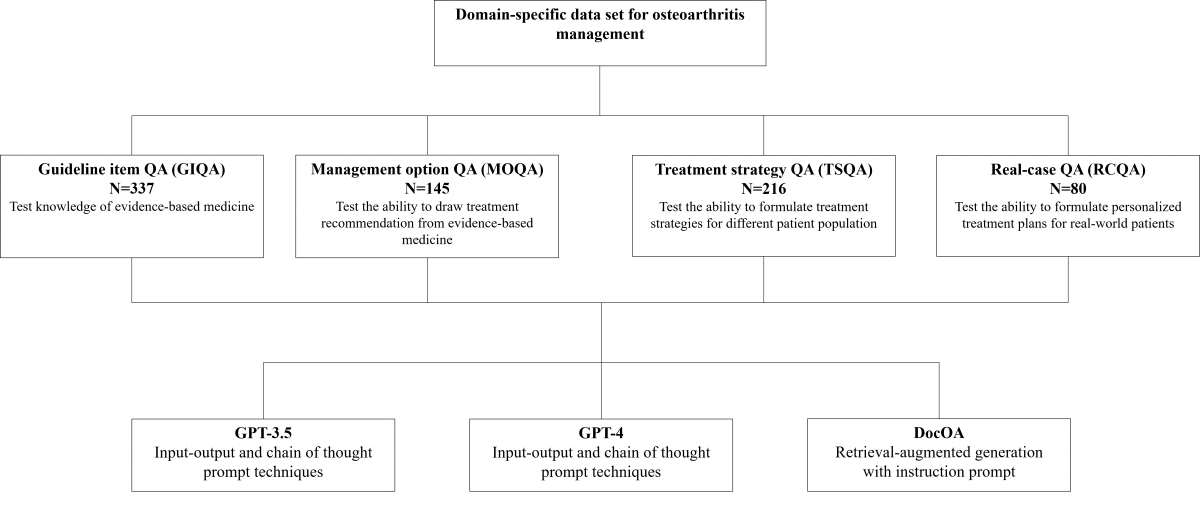
Study flow diagram. GIQA: guideline-item question-answer; MOQA: management options question-answer; QA: question-answer; real-case QA; TSQA: treatment strategy QA.

This study curated an OA management data set based on clinical guidelines and real-world cases. A benchmark was developed to evaluate the clinical knowledge and capabilities of LLMs for OA management. DocOA was built with instruction prompts and RAG and was tested along with other LLMs. Figure 1 illustrates the flow diagram of the study.

### Data Set

This data set was developed based on key clinical guidelines and real-world patients. After the panel discussion, 6 well-acknowledged guidelines and data from 80 real-world patients were selected that included various aspects of OA management. The following guidelines were included: American Academy of Orthopedic Surgeons management of OA of the knee (Nonarthroplasty) [13]; NICE (National Institute for Health and Care Excellence) guideline for OA in over 16s [14]; Osteoarthritis Research Society International (OARSI) guidelines for the nonsurgical management of knee, hip, and polyarticular OA [15]; Royal Australian College of General Practitioners Guideline for the management of knee and hip OA [16]; American College of Rheumatology/Arthritis Foundation (ACR) Guideline for the Management of Osteoarthritis of the Hand, Hip, and Knee [17]; European League Against Rheumatism (EULAR) recommendations for the nonpharmacological core management of hip and knee OA [18]. Between April 1, 2023, and October 1, 2023, a total of 80 patients diagnosed with OA and who had received OA management at our hospital were randomly selected. The patient information, including age, sex, height, weight, BMI, laterality of knee involvement, medical history, level of pain, mechanical symptoms, physical examination results, and radiographic findings, was retrieved. All identifiable information was concealed to maintain confidentiality.

The OA benchmark aims to test the clinical capabilities of LLMs at 4 levels within the context of evidence-based medicine, ranging from domain-specific knowledge to clinical capabilities. The benchmark assesses the performance of LLMs pertaining to OA knowledge, summarizing the knowledge to formulate recommendations for specific management options, providing tailored management options for different patient populations, and formulating personalized management plans for real-world cases.

### Assistant With RAG and Instruction Prompting

DocOA, a specialized assistant, was developed based on the GPT-4-1106-preview model, which integrates instruction prompts and RAG to enhance performance. The instruction prompt emphasized its role in providing evidence-based medical insights and personalized management programs guided by evidence-based medicine. The DocOA strictly adheres to facts, avoids speculation, and clearly states its limitations. Moreover, it maintains a professional and informative tone suitable for medical discussions.

**Figure 2:**
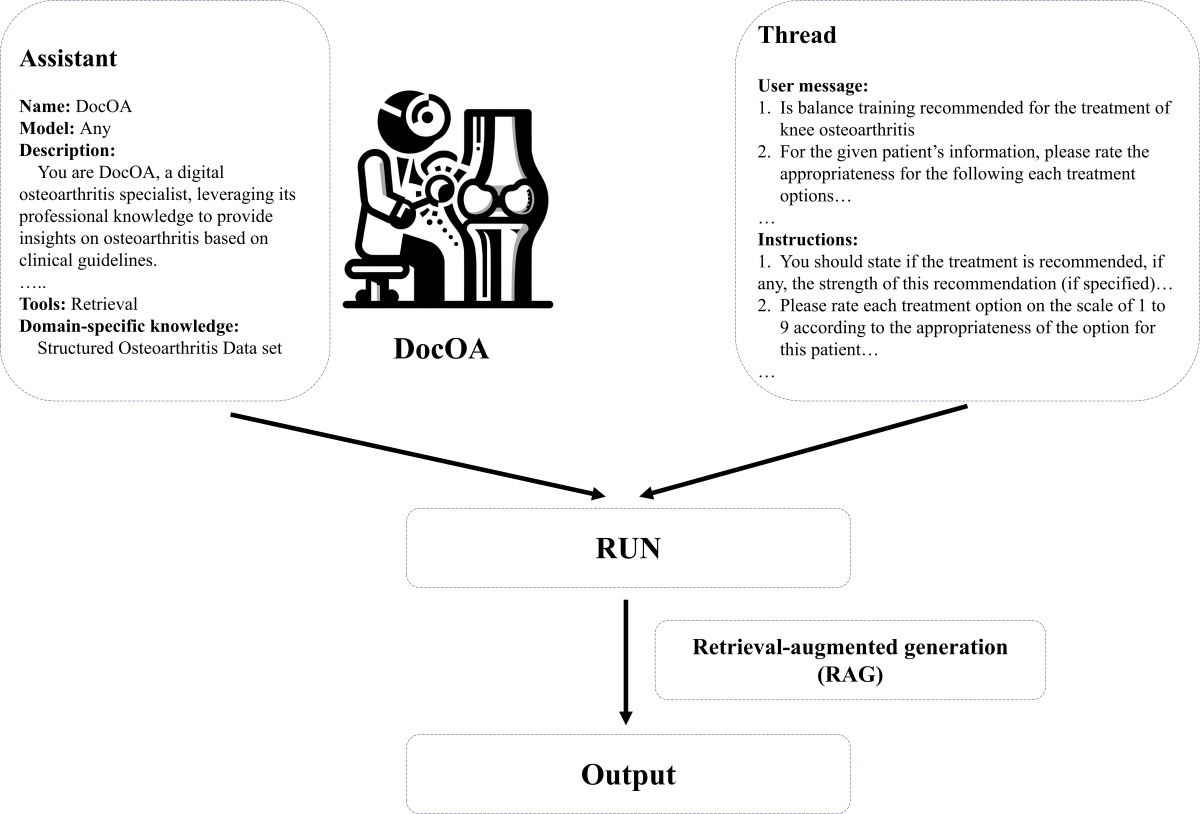
Workflow.

RAG has been used to respond to various OA-related queries. The RAG integrates a model's language generation capabilities with a retrieval system, enabling access to specific information from external sources [19]. Of the several RAG techniques and data structures tested, the retrieval function from OpenAI was adopted, and the most optimal data structure was selected and converted into the JSON format for optimal retrieval accuracy. In response to OA-related queries, the RAG enables the assistant to dynamically pull relevant data from the external data set as it generates responses. The workflow of the assistant is illustrated in Figure 2.

The assistant block details the core of the DocOA system, using a base model of GPT-4-1106-preview. The description within the block serves as an instruction prompt that outlines the system's role. The assistant’s functionality includes a retrieval tool that accesses external data set.

The system receives input from thread blocks in the form of questions about OA management and requests evaluation. This includes user messages with specific queries regarding OA treatment and detailed instructions for the system to follow.

The execution command then triggers the processing of input data through the DocOA system. Through RAG, DocOA incorporates external information from the knowledge database and follows the instructions to generate the final output. Simultaneously, based on instructional prompts, DocOA is capable of providing final outputs tailored to the user, whether they are patients or professional doctors, in the corresponding style.

### Models Testing

DocOA and the 2 base models, GPT-3.5 and GPT-4, were tested against the OA benchmark. Each question was presented 5 times to each model to assess the robustness of its performance. Additionally, the zero-shot chain of thoughts prompt technique was tested for GPT-3.5 and GPT-4 to determine whether it outperformed the input-output technique.

**Evaluation of LLMs’ Performance**

### Objective Evaluation

The model-generated responses were compared with predefined correct answers for each subset of the benchmark. An answer was considered accurate if LLM provided correct knowledge (recommendation status and recommendation strength) about the treatment option and predicted the correct treatment recommendation (treatment appropriateness) for a specific patient profile or individual patient.

The human evaluation framework is an effective approach for identifying the gap between LLMs and clinical experts [3]. In this study, human evaluation was performed by both physicians and patients. A total of 80 items from the OA benchmark were randomly selected for a detailed human evaluation framework.

A panel of 5 experienced physicians evaluated the outputs from LLMs. The sequence of answers was randomized and the generating models were anonymized to ensure that the evaluation was conducted without any knowledge of the model that generated them. Specifically, each evaluator independently assessed the sampled responses. All sampled responses were compiled into an Excel (Microsoft Corp) spreadsheet. The model labels corresponding to the responses were concealed. The evaluation metrics were established based on a previous study with modifications [3]. The physician assessed the quality of the responses in the following domains: inaccurate content, relevance, hallucinations, missing content, likelihood of possible harm, extent of possible harm, and possibility of bias. The ability of LLM to achieve correct comprehension, retrieval, and reasoning was assessed using the method described in a previous study [20]. Patient evaluation was conducted by assessing the user intent fulfillment and helpfulness of the content. The detailed descriptions of each human evaluation metric are provided in Multimedia Appendix 1.

### Statistical Analysis

All statistical analyses were performed using the SPSS 25.0 software (IBM Corp) and GraphPad Prism (version 8; GraphPad Software). Discontinuous data are expressed as incidence and rate and analyzed using the chi-square test for differences. A *P* value less than .05 indicated statistical significance.

### Ethical Considerations

Real-case QA (RCQA) includes treatment recommendations for 80 real cases. The study have obtained approval from the Medical Ethics Committee of Sichuan University (approval number: 2023; review number 2277). Participants were informed that the study would need to use relevant data including clinical symptoms, imaging reports and physical examination data, but they would not receive any intervention measures and their personal identifiable information was anonymized. This study does not involve compensation.

## Results

### OA Benchmark

The benchmark comprised 4 subsets of QA evaluations designed to test the performance of LLMs across a spectrum ranging from domain-specific knowledge to practical capability. Guideline-item QA (GIQA), which was developed based on specific items extracted from the clinical guidelines, evaluates the LLMs’ knowledge of these well-established standards. The GIQA comprised 337 items. Management options QA (MOQA) included summarized recommendations for specific treatments from the included clinical guidelines. The MOQA, which comprised 145 items, evaluated LLMs’ knowledge of specific treatment options as well as their ability to summarize medical evidence. Treatment strategy QA (TSQA), which included treatment recommendations for different patient populations, provided treatment recommendations based on the patient’s age, clinical presentation, and other factors. The TSQA, which comprised 216 items, evaluated the capability of LLMs to derive treatment recommendations for specific patient types. RCQA included treatment recommendations for 80 real-world patients. The RCQA, which comprised 80 items, evaluated LLMs’ capability in formulating treatment recommendations in a more complicated scenario in which individual information is provided, mirroring real-world clinical decision-making. This data set is available on GitHub [21]. Examples of each QA type are shown in Multimedia Appendix 2.

### Objective Evaluation

**Figure 3:**
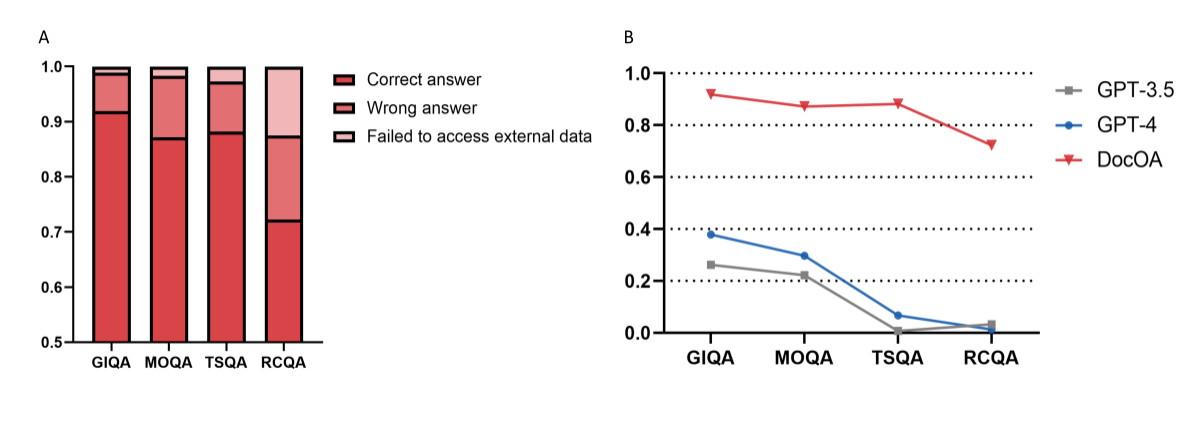
Results of objective evaluation. (A) Accuracy of each model against each subset of benchmark. (B) Inaccuracy analysis for DocOA due to wrong answer and failure to access external data. GIQA: guideline item question-answer (QA); MOQA: management option QA; TSQA: treatment strategy QA; RCQA: real-case QA.

The accuracy of GPT-3.5 in GIQA, MOQA, TSQA, and RCQA was 0.26, 0.22, 0.01, and 0.03, respectively. The accuracy of GPT-4 in GIQA, MOQA, TSQA, and RCQA was 0.38, 0.30, 0.07, and 0.01, respectively. The accuracy of DocOA in GIQA, MOQA, TSQA, and RCQA was 0.92, 0.87, 0.88, and 0.72, respectively. The accuracy of each model against the benchmark is presented in Table 1 and Figure 3A. As shown in Figure 3A, the degree of accuracy significantly decreased: GIQA˃MOQA˃TSQA˃RCQA. As shown in Figure 3B, DocOA reported 111 failures in accessing the external data set, which accounted for 12.4% of the inaccurate answers generated.

**Table 1:** Accuracy of each model against the osteoarthritis benchmark.

	GPT-3.5	GPT-4	DocOA	*P* value
Osteoarthritis benchmark	0.16	0.24	0.88	<.001^b^
Guideline item QA^a^	0.26	0.38	0.92	<.001^b^
Management option QA	0.22	0.30	0.87	<.001^b^
Treatment strategy QA	0.01	0.07	0.88	<.001^b^
Real-case QA	0.03	0.01	0.72	<.001^c^

^a^QA: question-answer.

^b^Further analysis showed all pairwise comparisons had a *P* value less than .05.

^c^Further analysis showed a *P* value is .056 for GPT-3.5 vs GPT-4, <.001 for GPT-3.5 vs DocOA, and <.001 for GPT-4 vs DocOA.

Zero-shot chain of thoughts prompt techniques were adopted for GPT-3.5 and GPT-4. Compared with the input-output prompt technique, no significant improvements in model performance were observed. The results are summarized in Table 2.

**Table 2:** Accuracy of different prompt techniques against the osteoarthritis benchmark.

	GPT-3.5			GPT-4		
	IO^a^	COT^b^	*P* value	IO	COT	*P* value
						
Osteoarthritis benchmark	0.16	0.17	.41	0.24	0.23	.52
Guideline item QA^c^	0.26	0.28	.03	0.38	0.38	.80
Management option QA	0.22	0.20	.004	0.30	0.27	.002
Treatment strategy QA	0.02	0.03	<.001	0.07	0.07	.79
Real-case QA	0.03	0.01	<.001	0.01	0.01	.20

^a^Input-output prompt technique.

^b^Zero-shot chain of thought prompt technique.

^c^QA: question-answer.

### Human Evaluation Results

From each of the GIQA, MOQA, TSQA, and RCQA, 20 items were randomly selected along with the corresponding responses generated by each model. A total of 1200 outputs were evaluated by physicians and patients. The results of the human evaluations of GPT-3.5, GPT-4, and DocOA revealed distinct outcomes across several aspects. A few examples have been selected and summarized in Multimedia Appendix 3, demonstrating how to apply the human evaluation framework to assess sampled responses.

Multimedia Appendix 4 shows the human evaluation results for the models’ output. The rate of inaccuracy was the highest for GPT-3.5 (57%, n=1200), followed by GPT-4 and DocOA at 50% (n=1200) and 19.3% (n=1200), respectively. All the models achieved high relevance and infrequently produced hallucinatory content in their responses. GPT-3.5 had a higher proportion of responses with missing content (22%, n=1200) than GPT-4 (16.4%, n=1200) or DocOA (16.5%, n=1200). GPT-3.5 presented a higher likelihood of generating harmful content (20%, n=1200) than GPT-4 (11.3%, n=1200) and DocOA (8.3%, n=1200). Moreover, GPT-3.5 was associated with a higher risk of causing severe harm (10.5%, n=1200) than GPT-4 (5.5%, n=1200) and DocOA (3.5%, n=1200). The likelihoods of potentially biased content were 13.3% (n=1200), 9.5% (n=1200), and 2.8% (n=1200) for GPT-3.5, GPT-4, and DocOA, respectively. The results of the human evaluation for each subset benchmark are listed in Multimedia Appendices 5-7. The results showed a substantial decrease in performance in terms of inaccurate content and missing content (GIQA˃MOQA˃TSQA˃RCQA).

**Figure 4:**
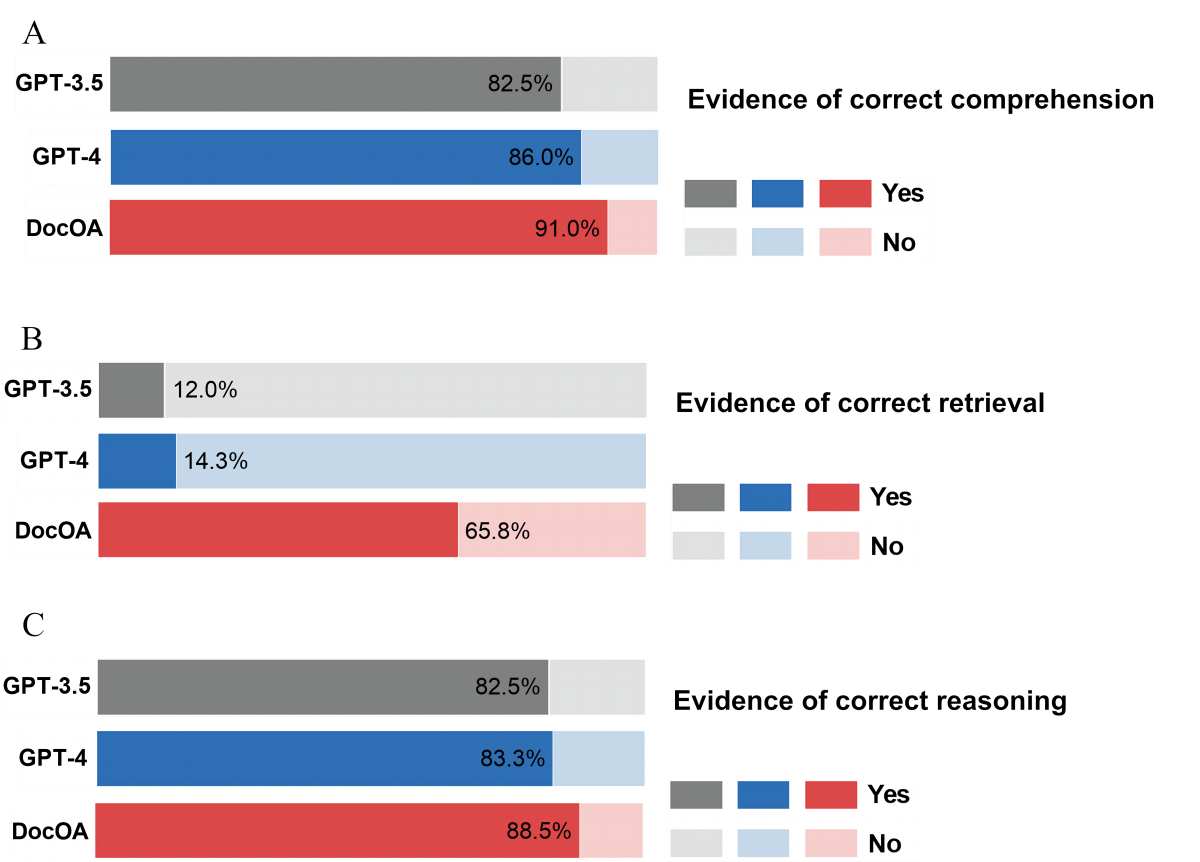
Results of human evaluation for LLMs’ comprehension, retrieval, and reasoning ability. (A) Evidence of correct comprehension. (B) Evidence of correct retrieval. (C) Evidence of correct reasoning.

Figure 4 shows the results of the LLMs’ ability to assess correct comprehension, correct retrieval, and correct reasoning. Regarding the correct comprehension of the question, the response rate of DocOA was 91% (n=1200), followed by GPT-4 (86%, n=1200) and GPT-3.5 (82.5%, n=1200). DocOA was able to correctly recall and present complete, relevant information in 65.8% (n=1200) of the responses, followed by GPT-4 (14.3%, n=1200) and GPT-3.5 (12.0%, n=1200). In terms of subset evaluation, the results showed comparable performance in comprehension and reasoning among the different models, whereas a substantial performance decrease was found in correct retrieval across GIQA, MOQA, TSQA, and RCQA.

**Figure 5:**
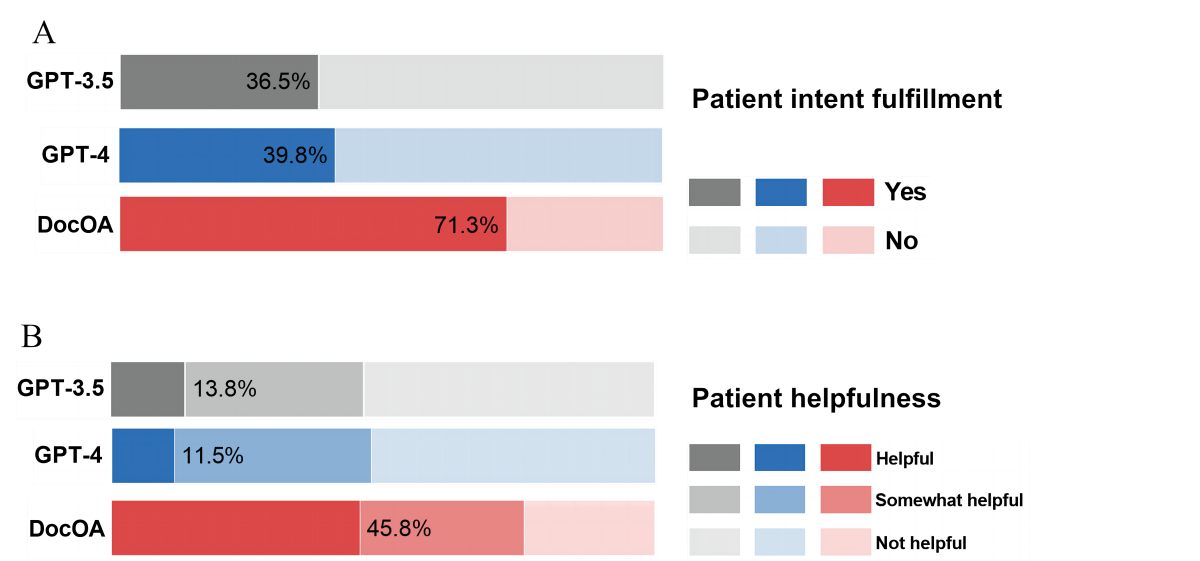
Results of human evaluation by patients. (A) Patient intent fulfillment. (B) Patient helpfulness.

The results of patient evaluations are shown in Figure 5. DocOA achieved a success rate of 71.3% in fulfilling patient intention, with GPT-4 at 39.8% (n=1200) and GPT-3.5 at 36.5% (n=1200). Of the responses generated by DocOA, 75.8% (n=1200) were considered to be at least somewhat helpful, compared to 47% (n=1200) for GPT-3.5 and 47.75% (n=1200) for GPT-4. For GPT-3.5 and GPT-4, the subset evaluation showed a substantial decrease in intent fulfillment and helpfulness as the tasks shifted from domain-specific knowledge to personalized treatment recommendations.

## Discussion

This study introduced a benchmark framework to assess the performance of LLMs in specific medical domains. Using OA as a case study, this framework is the first to evaluate LLMs across a spectrum, from domain-specific knowledge to clinical applications in specific disease management. The incorporation of human evaluation provides multiple dimensions of assessment that are of considerable interest to clinical practitioners and patients, making it an essential tool for evaluating the clinical capabilities of LLMs.

The study found that the integration of RAG and instructional prompts substantially improved the domain-specific capabilities and explainability of general-purpose LLMs without additional training. By contrast, general-purpose models such as GPT-3.5 and GPT-4 exhibit unsatisfactory performance when benchmarked against OA management. Additionally, both models demonstrated a marked decline in performance as tasks shifted from domain-specific knowledge to personalized treatment recommendations. Overall, the findings of this study demonstrate a cost-effective method for evaluating and enhancing the capabilities of LLMs in specialized medical fields.

### Domain-Specific Medical Benchmark

Although benchmarks targeting general medical knowledge have been previously developed [22-24], recent research has suggested that these benchmarks are only preliminary indicators of medical knowledge. The absence of tailored benchmarks in specific domains remains a potential challenge for evaluating the clinical effectiveness of LLMs [4,25]. Therefore, we developed a domain-specific benchmark focused on disease management for OA, which was selected for its prevalence, substantial disease burden, and complexity of its management strategies [6,7]. This benchmark was designed to test the domain-specific knowledge and clinical capabilities of LLMs. The benchmark comprised 4 parts, each testing the ability of LLMs at different levels, including the ability to provide evidence-based knowledge, summarizing knowledge to formulate recommendations, providing management recommendations for different patient populations, and formulating personalized management plans for real-world patients. The benchmark was constructed based on established clinical guidelines and real-world patient information. Clinical guidelines offer comprehensive reviews of updated evidence and expert opinions, making them reliable sources of domain-specific medical knowledge. Through panel discussions involving physicians and data scientists, the questions were designed in a hybrid format, integrating both definitive and interpretative elements. Using this benchmark, we confirmed that general-purpose LLMs exhibit suboptimal performance in specialized domains. A significant performance gap was observed between domain-specific knowledge and clinical proficiency. This highlights the challenges faced by general-purpose LLMs in effectively applying specialized knowledge to clinical scenarios.

### Human Evaluation Framework

Human evaluation is a crucial component in assessing the medical capabilities of LLMs and offers a multidimensional assessment of their clinical capabilities. In this study, the human evaluation framework was modified based on a previous study, and hallucinations and relevance were added as additional criteria [3,4]. The evaluation criteria included accuracy, relevance, hallucinations, omissions, potential harm, and biased content. Moreover, the performance of LLMs in question comprehension, information retrieval, and medical reasoning was evaluated, as these are crucial abilities in tailoring patient-specific treatment. Patient evaluations primarily determine how responses address the user’s intent and helpfulness. Although previous studies indicate a notable gap between objective benchmarking and human evaluation, our findings reveal a smaller discrepancy [3]. This could be attributed to the different knowledge domains and designs of the QA structure in this benchmark. The results of our study suggest that the GPT-3.5, GPT-4, and DocOA performed well in terms of hallucinations, comprehension, reasoning, and relevance. DocOA outperformed the other models in terms of accurate information, correct retrieval, and helpfulness as perceived by patients. This indicates that although generalized models are proficient in some areas, they remain inadequate in delivering the qualified responses required in a clinical context.

### Augmenting LLM With Domain-Specific Ability

Several techniques are available for developing medical LLMs, which primarily include integrating domain-specific knowledge during the training phase through techniques such as reinforcement learning with human feedback [26-28]. However, in this study, we focused on enhancing already-trained LLMs, such as GPT-4, by using a suite of techniques, including RAG and instruction-based prompts. Similar methodologies have been applied to the development of specialized LLMs for chemical domains [29].

This approach was adopted for the following reasons: first, augmenting an existing model such as GPT-4 is more cost-effective than training a new model from scratch; second, advanced general-purpose models have been trained on diverse data sets, providing a broad base of general knowledge that can be beneficial for understanding and contextualizing domain-specific information; third, techniques such as RAG and specialized prompting offer the convenience of being adjustable and refined over time, enabling easy adaptability to new evidence in the fast-evolving field of medicine.

Among these reasons, the role of RAG in this study needs to be emphasized. Explainability has always been a problem to be addressed in the application of LLMs, and it is also one of the ethical considerations in their application in the medical field. RAG enables the model to identify the source on which the generated answer was based, which significantly improves the explainability of the model. In this study, the knowledge base was structured in a way that allows RAG to identify the clinical evidence that the answer was based on. When DocOA generates an OA-relevant answer, it identifies the evidence on which the answer is based and makes it clear for professionals to evaluate the rationale and accuracy of its response. In general, provided with reliable and professional sources, RAG significantly improved the explainability and accuracy of LLMs in medical care.

Our results demonstrated that GPT-4 can effectively acquire domain-specific knowledge and clinical capabilities in the management of OA through a combination of approaches, including RAG and instruction prompts. This strategy can also be applied cost-effectively to other medical domains.

Nonetheless, the efficacy of the RAG is contingent upon factors such as the size and quality of the data, retrieval techniques used, and the underlying architecture of the LLM in use [30]. The evaluation results showed that DocOA was able to correctly understand the clinical question and avoid hallucinations. The fundamental reason for the errors stems from the inherent limitations of the RAG technology, where the retrieved information may sometimes be incomplete or may not adequately address the question.

In RAG, the query process is the key determinant of the information retrieved from the external knowledge base. In the query process, text is converted into an array of floating points. The entire array corresponds to a point in an n-dimensional space, which is known as the text vector, also referred to as an embedding [31,32]. The distance between vectors, which corresponds to semantic similarity, can be calculated. Therefore, by calculating semantic similarity, the query process retrieves the information from the external knowledge base that is considered most relevant to the query posted by the LLM. However, this process is sometimes uncertain because the most appropriate answer does not necessarily yield the highest similarity score. For example, in GIQA, the question is, “Is Walking aids, assistive technology and adaptations at home and at work recommended for Physical Treatment of Knee osteoarthritis according to 2013 EULAR recommendations for the nonpharmacological core management of hip and knee osteoarthritis?” For this question, out of the 5 responses generated by LLM, 4 incorrectly state “recommend” when the correct answer should be “may be used.” The error occurred because only a portion of the information was retrieved. However, neither the complete information nor the key information, which contains the answer, was retrieved. More specifically, if the retrieved information was “The frequent use of assistive technology and the high satisfaction rates with its use indicate that walking aids, assistive technology, and adaptations are important and useful for people with hip or knee OA.” The model might be misled to generate the incorrect answer.

Generally, enhancing the effectiveness of RAG is an ongoing effort in the field of computer science, and the effectiveness of RAG is likely to keep increasing with the rapid development of LLMs and RAG technologies.

### The Potential Value of DocOA in Future Clinical Practice

**Figure 6:**
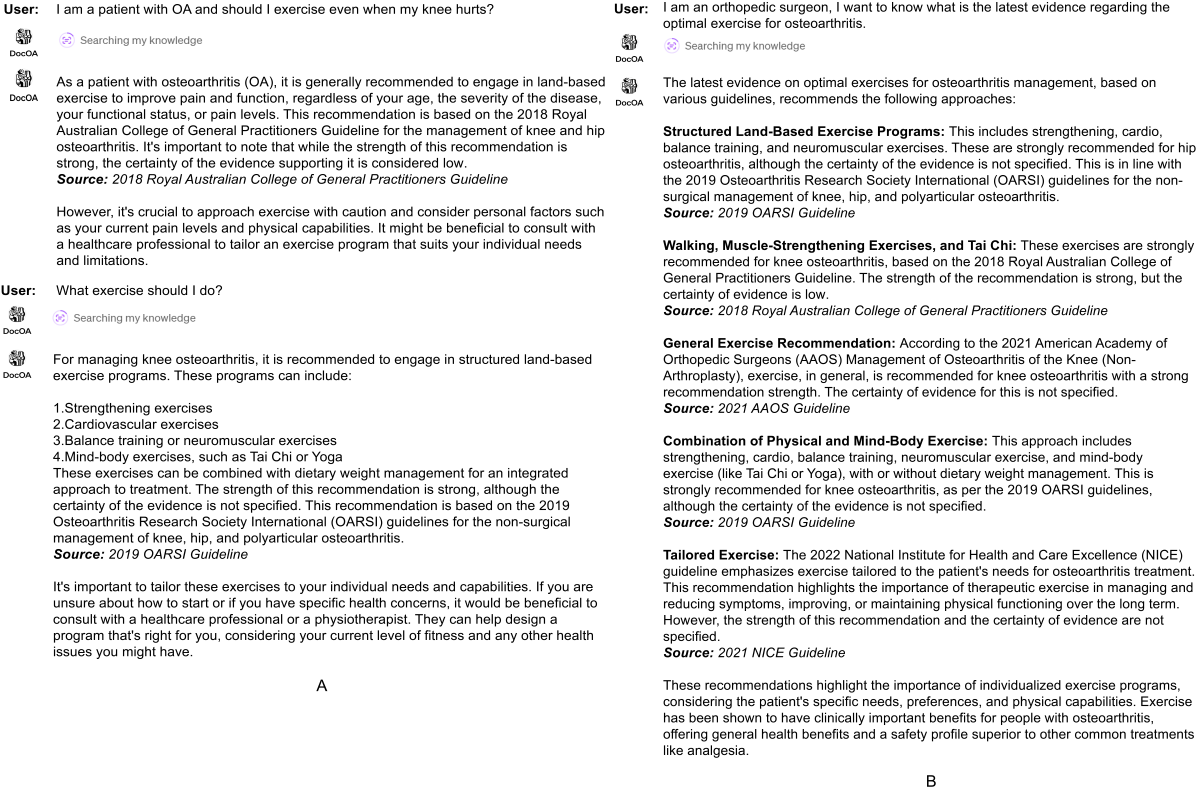
Examples of clinical practice. (A) How DocOA provides exercise guidance for patients with OA. (B) How DocOA provides doctors with clinical evidence for exercise guidance in OA, as well as the clinical evidence the answer is based on.

DocOA has shown promising potential in clinical practice due to its proficient OA management knowledge and the ability to explain its answer. Previous studies have shown general-purpose LLMs to be limited in medical specialties such as nephrology, ophthalmology, and OA. The versatile and unverified training data could contain misleading medical information, which led to inaccurate answers. On the other hand, DocOA has shown good results in providing evidence-based OA management knowledge and formulating personalized treatment plans. To adopt DocOA in clinical practice, it is designed to answer patient queries and doctor queries differently, as shown in Figure 6. When generating answers for medical professionals, it is able to generate answers based on selected high-quality clinical evidence and accurately identify the corresponding references, thus providing explainable and evidence-based answers.

### Limitations

This study has several potential limitations that need to be addressed. First, OA management is highly complex, and our current data set remains limited and requires continuous supplementation and updating. Therefore, establishing specialized groups dedicated to building and updating these LLM databases is imperative for diverse medical applications. Second, our reliance on English sources could restrict the applicability and inclusivity of our findings across different linguistic and cultural contexts. Third, although there have been human evaluations of clinical case data, the augmented model has not yet been tested in a real-world clinical setting. These limitations highlight the necessity for ongoing development and a comprehensive, multidimensional approach for evaluating LLMs in the medical field.

### Conclusions

In this study, we introduce a novel benchmark framework designed to evaluate the capabilities of LLMs in specific medical domains, with OA serving as a case study. This framework assesses LLMs in terms of medical knowledge, evidence summarization, and clinical capabilities. Through a combination of objective measures and human evaluations, we identified the limitations of generalized LLMs in clinical contexts. Furthermore, our study demonstrated that DocOA, which integrates RAG and instructional prompts, significantly improves both the domain-specific performance and the explainability of LLMs. This approach is a potentially cost-effective strategy for developing domain-specific medical LLMs.

## Appendix

Multimedia Appendix 1 Details of human evaluation framework.

Multimedia Appendix 2 Benchmark framework for osteoarthritis management.

Multimedia Appendix 3 Examples for human evaluation.

Multimedia Appendix 4 Results of human evaluation for the assessment of responses.

Multimedia Appendix 5 Human evaluation results for GPT-3.5 across GIQA, MOQA, TSQA and RCQA.

Multimedia Appendix 6 Human evaluation results for GPT-4 across GIQA, MOQA, TSQA and RCQA.

Multimedia Appendix 7 Human evaluation results for DocOA across GIQA, MOQA, TSQA and RCQA.

## Abbreviations

ACR: American College of Rheumatology/Arthritis Foundation
EULAR: European League Against Rheumatism
GIQA: guideline-item question-answer
LLM: large language model
MOQA: management options question-answer
NICE: National Institute for Health and Care Excellence
OA: osteoarthritis
OARSI: Osteoarthritis Research Society International
QA: question-answer
RAG: retrieval-augmented generation
RCQA: real-case QA
TSQA: treatment strategy QA

## Data Availability

The data sets generated during or analyzed during this study are available in the GitHub repository Osteoarthritis-Benchmark [21]. The data citation should include a persistent identifier (eg, web URL or DOI) cited per journal style in the reference list.
