# Supplementary material for: Evaluating and Enhancing Large Language Models’ Performance in Domain-Specific Medicine: Development and Usability Study With DocOA
Source: J Med Internet Res. 2024 Jul 22;26:e58158. doi: 10.2196/58158 (PMC11301122; doi:10.2196/58158)
Supplement: Multimedia Appendix 2 [file jmir_v26i1e58158_app2.pdf]

Guideline Item QA (GIQA)

Question: Is balance training recommended for the treatment of knee osteoarthritis according to 2019 American College of Rheumatology/Arthritis Foundation(ACR) Guideline for the Management of Osteoarthritis of the Hand, Hip, and Knee? What is the level of recommendation strength and level of evidence?

Answer:
Recommendation Status: Recommend
Recommendation Strength: Conditional
Long Answer:
Balance training is recommended for knee osteoarthritis treatment. The strength of recommendation is Conditional. The certainty of evidence is IIb(At least one type of quasi-experimental study). This answer is according to the 2019 American College of Rheumatology/Arthritis Foundation (ACR) Guideline for the Management of Osteoarthritis of the Hand, Hip, and Knee.
Balance exercises include those that improve the ability to control and stabilize body position
Although one might expect balance exercises to help reduce the risk of falls in patients with OA, RCTs to date have not addressed this outcome in this population, and the low quality of evidence addressing the use of balance exercises necessitates only a conditional recommendation for balance exercises

Treatment Strategy QA (TSQA)

Question:
For the given patient profile, please rate the appropriateness for each treatment option. The patients is middle-aged; The patient has function-limiting pain that is intermittent and predictable; The patient has arthritic involvement predominantly in one weight bearing compartment. Radiographs show minimal joint space narrowing (KL 0-1). In terms of mechanical symptoms suggesting loose body or meniscal tear, the patient has mechanical symptoms present.
The treatment options to be evaluated include: **1.**Self management program; **2.**Prescribed Physical Therapy; **3.**Hinged Knee Brace and/or Unloading Brace, Assistive Devices; **4.**NSAID or Acetaminophen; **5.**Arthroscopic Partial Meniscectomy or Shaving; **6.**Intraarticular Corticosteroids; **7.**Platelet rich plasma (PRP).

Answer:
The appropriateness of each treatment option for this type of patient profile is: **1.**Self-Management Programs (unsupervised exercise, tai chi, weight loss, aerobic walking) is Appropriate Rating: 8; **2.**Prescribed Physical Therapy (Supervised Exercise, manual therapy, neuromuscular training, etc.) is Appropriate Rating: 8; **3.**NSAID or Acetaminophen is Appropriate Rating: 8; **4.**Intraarticular Corticosteroids is Appropriate Rating: 8; **5.**Arthroscopic Partial Meniscectomy or Shaving is Appropriate Rating: 7; **6.**Hinged Knee Brace and/or Unloading Brace, Assistive Devices (e.g., cane, walker) is May Be Appropriate Rating: 6; **7.**PRP is Rarely Appropriate Rating: 3

Management Options QA (MOQA)

Question: Is lateral wedged insoles recommended for the treatment of knee osteoarthritis.

Answer:
Recommendation Status: Not Recommend
Recommendation Strength: Conditional/Strong
Long Answer:
Lateral wedged insoles is not recommended for the physical treatment of hip and knee osteoarthritis.
This recommendation is according to the 2019 American College of Rheumatology/Arthritis Foundation (ACR) Guideline for the Management of Osteoarthritis of the Hand, Hip, and Knee, the strength of recommendation is Conditional. The certainty of evidence is Ia (Meta-analysis of randomised controlled trials). This recommendation is also according to 2021 American Academy of Orthopedic Surgeons (AAOS) Management of Osteoarthritis of the Knee (Non-Arthroplasty). The strength of recommendation is Strong. The certainty of evidence is Not specified.
Lateral wedge insoles are higher on the lateral side (and may include a subtalar strapping component), shifting weight toward the lateral tibiofemoral compartment, and are applicable for those with medial compartment tibiofemoral OA and varus deformity. Shock-absorbing insoles are made of a material that aims to absorb impact loading during walking. Arch supports are insoles designed to support and realign the foot.
Explanation and Additional Information: truncated due to figure size

Real-Case QA (RCQA)

Question:
For the given patient's condition, please rate the appropriateness of the following treatment options. The patient is a 37 years old female who is experiencing discomfort in the bilateral knee. The patient is 165 cm in height and 60 kg in weight, the BMI is 22.03 kg/m2. The patient's past medical history is allergic to NSAIDs The patients has function-limiting pain that is constant with intense intermittent unpredictable episodes. The patient has arthritic Involvement predominantly in one weight bearing compartment. Imaging and physical examination revealed severe joint space narrowing (KL 4) with significantly limited range of motion. The patient has mechanical symptoms that is compatible with meniscal tear or loose body.

Answer:
For this patient, the appropriateness of each treatment option for this patient is as follows: **1.**Self-management programs (unsupervised exercise, tai chi, weight loss, aerobic walking) is Appropriate and the rating is 8. **2.**Prescribed physical therapy (supervised exercise, manual therapy, neuromuscular training, etc.) is Appropriate and the rating is 8. **3.**Hinged knee brace and/or unloading brace, assistive devices (e.g., cane, walker) is Appropriate and the rating is 7. **4.**NSAID or Acetaminophen is Not appropriate. **5.**Intraarticular corticosteroids is Appropriate and the rating is 9. **6.**Arthroscopic partial meniscectomy or shaving is Rarely appropriate and the rating is 3. **7.**Platelet rich plasma is Rarely appropriate and the rating is 3.

Supplementary Figure 1. Benchmark framework for osteoarthritis management
