# Supplementary material for: Evaluating and Enhancing Large Language Models’ Performance in Domain-Specific Medicine: Development and Usability Study With DocOA
Source: J Med Internet Res. 2024 Jul 22;26:e58158. doi: 10.2196/58158 (PMC11301122; doi:10.2196/58158)
Supplement: Multimedia Appendix 3 [file jmir_v26i1e58158_app3.pdf]

1. Inaccurate content

LLM's Answer（From GPT-3.5）

{
"Intervention": "Modified shoes",
"RecommendationStatus": "May be used",
"StrengthOfRecommendation": "Not specified",
"LevelOfCertainty": "Not specified",
"Source": "Clinical guidelines for the management of osteoarthritis",
"BriefExplanationAndAdditionalInformation": "Modified shoes may be used as part of a multimodal approach for the physical treatment of osteoarthritis. The use of modified shoes should be individualized based on patient needs and preferences, and may be considered in combination with other non-pharmacologic interventions such as exercise, physical therapy, and weight management."
}

Answer from external knowledge base:

Modified shoes is Not Recommend for knee osteoarthritis PhysicalTreatment or hip osteoarthritis PhysicalTreatment. The strength of recommendation is Conditional. The certainty of evidence is III (Descriptive studies, such as comparative studies, correlation studies or case–control studies). According to 2019 American College of Rheumatology/Arthritis Foundation(ACR) Guideline for the Management of Osteoarthritis of the Hand, Hip, and Knee.

Modifications to shoes can be intended to alter the biomechanics of the lower extremities and the gait. While optimal footwear is likely to be of considerable importance for those with knee and/or hip OA, the available studies do not define the best type of footwear to improve specific outcomes for knee or hip OA.

Human Evaluation:

The answer from the external knowledge base states that modified shoes are not recommended for physical treatment of knee or hip osteoarthritis. However, the LLM's answer suggests that the recommendation status is "May be used," which does not align. And the answer is considered inaccurate. Yet, since the content from the external knowledge base, which states 'While optimal footwear is likely to be of considerable importance for those with knee and/or hip OA, the available studies do not define the best type of footwear to improve specific outcomes for knee or hip OA,' corresponds with the content from the Brief Explanation and Additional Information, which states 'The use of modified shoes should be individualized based on patient needs and preferences, and may be considered in combination with other non-pharmacologic interventions such as exercise, physical therapy, and weight management,' it was not judged as 'Harmful.'

2.Omission

LLMs' Answer（From GPT-3.5）

{
  "Intervention": "Acupuncture (traditional, laser, electrical)",
  "RecommendationStatus": "Recommended",
  "StrengthOfRecommendation": "Strong",
  "LevelOfCertainty": "High",
  "Source": "American College of Rheumatology (ACR) guidelines"
}

Answer from external knowledge base:

Acupuncture is Recommend for Hand osteoarthritis Physical Treatment, Knee osteoarthritis Physical Treatment and Hip osteoarthritis Physical Treatment. The strength of recommendation is Conditional. The certainty of evidence is Ia (Meta-analysis of randomised controlled trials). According to 2019 American College of Rheumatology/Arthritis Foundation(ACR) Guideline for the Management of Osteoarthritis of the Hand, Hip, and Knee.

Acupuncture is Not recommended for osteoarthritis PhysicalTreatment. The strength of recommendation is Not specified. The certainty of evidence is Not specified. According to 2022 National Institute for Health and Care Excellence (NICE) guideline for osteoarthritis in over 16s: diagnosis and management.

Human Evaluation:

The answer from the external knowledge base included two sources: the 2022 National Institute for Health and Care Excellence (NICE) guideline for osteoarthritis in over 16s: diagnosis and management, and the 2019 American College of Rheumatology/Arthritis Foundation (ACR) Guideline for the Management of Osteoarthritis of the Hand, Hip, and Knee. However, the LLM's answer included only one of these, and therefore it was judged as an omission.

3.Inaccurate and Harmful

LLM's Answer（From GPT-3.5）

{
  "Intervention": "Pulsed vibration therapy",
  "RecommendationStatus": "Recommended",
  "StrengthOfRecommendation": "Strong",
  "LevelOfCertainty": "High",
  "Source": "Osteoarthritis Research Society International (OARSI) guidelines"
}

Answer from external knowledge base:

Pulsed vibration therapy is Not Recommend for knee osteoarthritis PhysicalTreatment. The strength of recommendation is Conditional. The certainty of evidence is IIb(At least one type of quasi-experimental study). According to 2019 American College of Rheumatology/Arthritis Foundation(ACR) Guideline for the Management of Osteoarthritis of the Hand, Hip, and Knee.

Pulsed vibration therapy is Not Recommend for knee osteoarthritis PhysicalTreatment. The strength of recommendation is Conditional. The certainty of evidence is IIb(At least one type of quasi-experimental study). According to 2019 American College of Rheumatology/Arthritis Foundation(ACR) Guideline for the Management of Osteoarthritis of the Hand, Hip, and Knee.

Few trials have addressed pulsed vibration therapy, and in the absence of adequate data, we conditionally recommend against its use

Human Evaluation:

The answer from external knowledge base serves as correct answer, which states that pulsed vibration therapy is 'not recommended', whereas the answer generated by the LLM is 'recommended', which is completely opposite. This response is considered 'inaccurate. Given the potential risk of side effect associated with pulsed vibration therapy, the answer is also considered harmful. Additionally, the evidence provided by the model is incorrectly sourced. The model attributes the information to the Osteoarthritis Research Society International (OARSI) guidelines, which is inaccurate. In fact, the source of this evidence is the 2019 American College of Rheumatology/Arthritis Foundation (ACR) Guideline for the Management of Osteoarthritis of the Hand, Hip, and Knee.
