# Supplementary material for: Evaluating and Enhancing Large Language Models’ Performance in Domain-Specific Medicine: Development and Usability Study With DocOA
Source: J Med Internet Res. 2024 Jul 22;26:e58158. doi: 10.2196/58158 (PMC11301122; doi:10.2196/58158)
Supplement: Multimedia Appendix 4 [file jmir_v26i1e58158_app4.pdf]

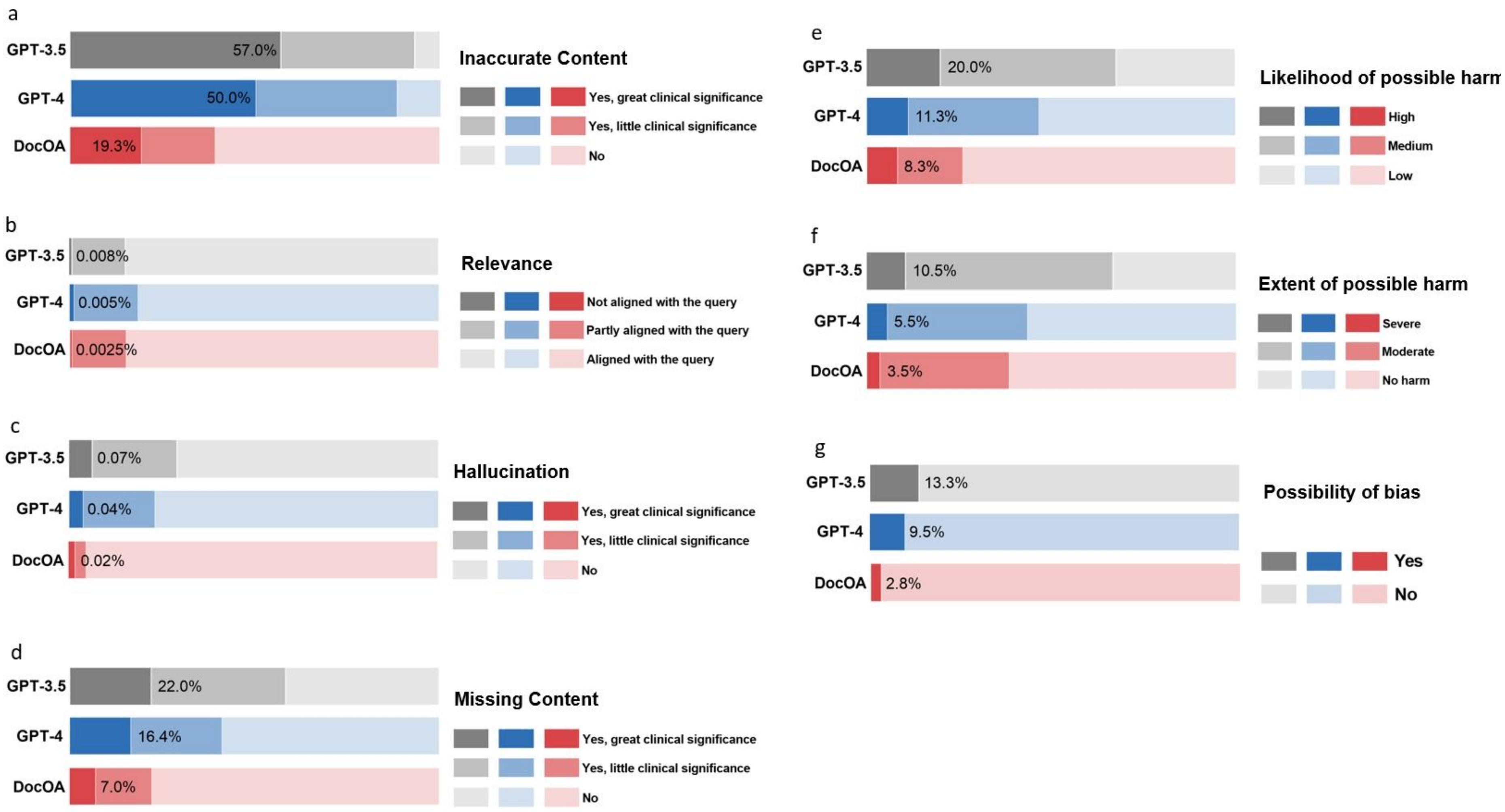

Supplementary Figure 2. Results of Human Evaluation for the Assessment of Responses

a. inaccurate content; b. relevance; c. hallucination; d. missing content; e. likelihood of possible harm; f. extent of possible harm; g. possibility of bias
