# Supplementary material for: Evaluating and Enhancing Large Language Models’ Performance in Domain-Specific Medicine: Development and Usability Study With DocOA
Source: J Med Internet Res. 2024 Jul 22;26:e58158. doi: 10.2196/58158 (PMC11301122; doi:10.2196/58158)
Supplement: Multimedia Appendix 5 [file jmir_v26i1e58158_app5.pdf]

Supplementary file 3 Human evaluation results for GPT-3.5 across GIQA, MOQA, TSQA and RCQA

		Inaccurate content			Relevance			Hallucination		
		Yes, great clinical significance	Yes, little clinical significance	No	Not aligned with the query	Partly aligned with the query	Aligned with the query	Yes, great clinical significance	Yes, little clinical significance	No
GPT-3.5	GIQA	29%	61%	10%	0%	9%	91%	4%	16%	80%
	MOQA	37%	48%	15%	0%	22%	78%	4%	33%	63%
	TSQA	82%	18%	0%	0%	12%	88%	11%	23%	66%
	RCQA	80%	18%	2%	3%	15%	82%	6%	20%	74%
	Total	57%	36.25%	6.75%	75%	14.5%	84.75%	6.25%	23%	78%

		Missing Content			Likelihood of Possible Harm			Extent of Possible Harm		
		Yes, great clinical significance	Yes, little clinical significance	No	High	Medium	Low	Severe	Moderate	No harm
GPT-3.5	GIQA	22%	61%	17%	17%	52%	31%	8%	47%	45%
	MOQA	14%	42%	44%	17%	48%	35%	9%	32%	59%
	TSQA	33%	23%	44%	25%	41%	34%	14%	70%	16%
	RCQA	19%	20%	61%	21%	50%	29%	11%	76%	13%
	Total	22%	36.5%	41.5%	20%	47.75%	32.25%	15%	56.25%	33.3%

		Possibility of Bias	
		Yes	No
GPT-3.5	GIQA	9%	91%
	MOQA	19%	81%
	TSQA	14%	86%
	RCQA	11%	89%
	Total	13.3%	86.8%

		Correct Comprehension		Correct Retrieval		Correct Reasoning	
		Yes	No	Yes	No	Yes	No
GPT-3.5	GIQA	76%	24%	20%	80%	88%	12%
	MOQA	81%	19%	17%	83%	81%	19%
	TSQA	92%	8%	3%	97%	79%	21%
	RCQA	81%	19%	8%	92%	82%	18%
	Total	82.5%	17.5%	12%	88%	82.5%	17.5%

		User Intent Fulfillment		User Helpfulness		
		Yes	No	Helpful	Somewhat helpful	Not helpful
GPT-3.5	GIQA	59%	41%	7%	41%	52%
	MOQA	40%	60%	18%	40%	42%
	TSQA	26%	74%	8%	23%	69%
	RCQA	21%	79%	22%	25%	53%
	Total	36.5%	63.5%	13.75%	32.25%	54%
