# Supplementary material for: Evaluating and Enhancing Large Language Models’ Performance in Domain-Specific Medicine: Development and Usability Study With DocOA
Source: J Med Internet Res. 2024 Jul 22;26:e58158. doi: 10.2196/58158 (PMC11301122; doi:10.2196/58158)
Supplement: Multimedia Appendix 6 [file jmir_v26i1e58158_app6.pdf]

Supplementary file 4 Human evaluation results for GPT-4 across GIQA, MOQA, TSQA and RCQA

		Inaccurate content			Relevance			Hallucination		
		Yes, great clinical significance	Yes, little clinical significance	No	Not aligned with the query	Partly aligned with the query	Aligned with the query	Yes, great clinical significance	Yes, little clinical significance	No
GPT-4	GIQA	29%	55%	16%	0%	16%	84%	2%	17%	81%
	MOQA	38%	40%	22%	1%	19%	80%	5%	22%	73%
	TSQA	76%	20%	4%	0%	13%	87%	8%	18%	74%
	RCQA	57%	38%	5%	1%	11%	88%	0%	21%	79%
	Total	50%	38.25%	11.75%	0.5%	14.75%	84.75%	3.75%	19.5%	76.75%

		Missing Content			Likelihood of Possible Harm			Extent of Possible Harm		
		Yes, great clinical significance	Yes, little clinical significance	No	High	Medium	Low	Severe	Moderate	No harm
GPT-4	GIQA	7%	37%	56%	2%	39%	59%	3%	25%	72%
	MOQA	20%	31%	49%	8%	32%	60%	6%	17%	77%
	TSQA	29%	20%	51%	22%	32%	46%	10%	62%	28%
	RCQA	10%	11%	79%	13%	39%	48%	3%	48%	49%
	Total	16.5%	24.75%	58.75%	11.25%	35.5%	53.25%	5.5%	38%	56.5%

		Possibility of Bias	
		Yes	No
GPT-4	GIQA	5%	95%
	MOQA	15%	85%
	TSQA	8%	92%
	RCQA	10%	90%
	Total	9.5%	90.5%

		Correct Comprehension		Correct Retrieval		Correct Reasoning	
		Yes	No	Yes	No	Yes	No
GPT-4	GIQA	94%	6%	19%	81%	87%	13%
	MOQA	74%	26%	27%	73%	80%	20%
	TSQA	94%	6%	6%	94%	85%	15%
	RCQA	82%	18%	5%	95%	81%	19%
	Total	86%	14%	14.25%	85.75%	83.25%	16.75%

		User Intent Fulfillment		User Helpfulness		
		Yes	No	Helpful	Somewhat helpful	Not helpful
GPT-4	GIQA	63%	37%	13%	38%	49%
	MOQA	33%	67%	16%	32%	52%
	TSQA	28%	72%	6%	33%	61%
	RCQA	35%	65%	11%	42%	47%
	Total	39.75%	60.25%	11.5%	36.25%	52.25%
