# Supplementary material for: Evaluating and Enhancing Large Language Models’ Performance in Domain-Specific Medicine: Development and Usability Study With DocOA
Source: J Med Internet Res. 2024 Jul 22;26:e58158. doi: 10.2196/58158 (PMC11301122; doi:10.2196/58158)
Supplement: Multimedia Appendix 7 [file jmir_v26i1e58158_app7.pdf]

Supplementary file 5 Human evaluation results for DocOA across GIQA, MOQA, TSQA and RCQA

`		Inaccurate content			Relevance			Hallucination		
		Yes, great clinical significance	Yes, little clinical significance	No	Not aligned with the query	Partly aligned with the query	Aligned with the query	Yes, great clinical significance	Yes, little clinical significance	No
Retrieval	GIQA	8%	18%	74%	0%	9%	91%	2%	7%	91%
	MOQA	8%	14%	78%	0%	10%	90%	1%	11%	88%
	TSQA	4%	10%	86%	0%	8%	92%	4%	6%	90%
	RCQA	57%	38%	5%	1%	11%	88%	0%	21%	79%
	Total	19.25%	20%	60.75%	0.25%	9.5%	90.25%	1.75%	11.25%	87%

		Missing Content			Likelihood of Possible Harm			Extent of Possible Harm		
		Yes, great clinical significance	Yes, little clinical significance	No	High	Medium	Low	Severe	Moderate	No harm
Retrieval	GIQA	3%	25%	72%	7%	10%	83%	0%	17%	83%
	MOQA	7%	12%	81%	4%	11%	85%	1%	13%	86%
	TSQA	8%	13%	79%	9%	11%	80%	10%	62%	28%
	RCQA	10%	11%	79%	13%	39%	48%	3%	48%	49%
	Total	7%	15.25%	77.75%	8.25%	17.75%	74%	35%	35%	61.5%

		Possibility of Bias	
		Yes	No
Retrieval	GIQA	25%	75%
	MOQA	8%	92%
	TSQA	8%	92%
	RCQA	10%	90%
	Total	12.75%	87.25%

		Correct Comprehension		Correct Retrieval		Correct Reasoning	
		Yes	No	Yes	No	Yes	No
Retrieval	GIQA	100%	0%	86%	14%	96%	04%
	MOQA	89%	11%	88%	12%	90%	10%
	TSQA	93%	7%	84%	16%	87%	13%
	RCQA	82%	18%	5%	95%	81%	19%
	Total	91%	9%	65.75%	34.25%	88.5%	11.5%

		User Intent Fulfillment		User Helpfulness		
		Yes	No	Helpful	Somewhat helpful	Not helpful
Retrieval	GIQA	93%	7%	64%	26%	10%
	MOQA	89%	11%	66%	23%	11%
	TSQA	68%	32%	42%	30%	28%
	RCQA	35%	65%	11%	42%	47%
	Total	71.25%	28.75%	45.75%	30.25%	24%
